# When “Self‐Harm” Means “Suicide”: A Topic Modeling Study of Adolescent Online Help‐Seeking for Self‐Harm

**DOI:** 10.1111/sltb.70055

**Published:** 2025-11-17

**Authors:** Monika Neff Lind, Afsaneh Razi, Hanneke Scholten, Madeleine J. George, Munmun De Choudhury, Isabela Granic, Shalini Lal, Pamela J. Wisniewski, Nicholas B. Allen

**Affiliations:** ^1^ Department of Psychology University of Oregon Eugene Oregon USA; ^2^ Department of Psychological Science University of California Irvine California USA; ^3^ Department of Information Science Drexel University Philadelphia Pennsylvania USA; ^4^ Department of Computer Science University of Central Florida Orlando Florida USA; ^5^ Department of Child and Adolescent Psychiatry/Psychology Erasmus Medical Center Rotterdam the Netherlands; ^6^ Community Health and Implementation Research RTI International Research Triangle Park North Carolina USA; ^7^ School of Interactive Computing Georgia Institute of Technology Atlanta Georgia USA; ^8^ Faculty of Social Sciences McMaster University Hamilton Ontario Canada; ^9^ School of Rehabilitation University of Montreal Montreal Quebec Canada; ^10^ International Computer Science Institute Berkeley California USA

**Keywords:** adolescence, help‐seeking, online, peer support, self‐harm, suicide

## Abstract

**Introduction:**

The 15%–20% of adolescents worldwide who engage in nonsuicidal self‐injury (NSSI) face an increased risk of transitioning from suicidal ideation to suicide attempt. To resist NSSI urges, young people often seek peer support online. We examined adolescent help‐seeking on a purpose‐built online mental health peer support platform, which is a critically understudied help‐seeking venue.

**Methods:**

Adolescents' help‐seeking posts in the “Self Harm” category on a large online peer support platform (575,261 posts from 114,937 users) were analyzed using topic modeling. We assessed the prevalence of NSSI‐related topics versus morbid/suicidal topics.

**Results:**

Our 12‐topic model produced interpretable themes. Three main findings emerged: posts included little information about the context of self‐harm behavior; there was minimal evidence of pro‐self‐harm content in posts; and the primary topics of the posts were evenly split between NSSI‐related topics and morbid/suicidal topics.

**Conclusion:**

Our findings have important implications for online mental health communities: requiring users to select a narrow category for their post may limit contextual information; moderation of pro‐self‐harm content may reduce its prevalence; and the absence of dedicated spaces for suicidal users may funnel those users into NSSI‐focused spaces, potentially increasing risk for all users.

Nonsuicidal self‐injury (NSSI) in adolescence is common and risky. Around the world, adolescent NSSI occurs at prevalence rates of 15%–20% (Muehlenkamp et al. 2012). This behavior is a compelling target for psychological research not only because of the harm that it causes, but also because of its seeming rejection of core self‐preservation instincts (Hooley and Franklin 2018). Much of the research and clinical interest in NSSI concerns its relationship to suicide, the second leading cause of death in American teens (Shain et al. 2016). Nonsuicidal self‐injury is associated with suicidal thoughts and behaviors over and above the effect of common mental disorders, and NSSI is associated with increased risk of transitioning from suicidal ideation to suicide attempt (Kiekens et al. 2018). In short, reducing NSSI could reduce deaths by suicide. In this study, we support efforts to reduce NSSI by studying help‐seeking on a purpose‐built online mental health peer support platform in contrast to most other studies, which focus on general use platforms like Reddit. This contrast allows us to shine a light on a critically understudied venue for online help‐seeking (i.e., bespoke mental health platforms) and provide specific, actionable insights for online efforts to reduce NSSI.

Numerous barriers have impeded the progress of programs that aim to reduce NSSI. Clinician‐delivered, evidence‐based treatments for NSSI show minimal or mixed effectiveness (for excellent summaries, see: Dobias et al. 2021; Preston and West 2022). Stigma can also prevent people with NSSI from seeking professional help or in‐person peer support (Lavis and Winter 2020). Those who do seek help often encounter service gaps, such as long waiting lists that occur both in person and online (Lavis and Winter 2020).

In light of these barriers, people with NSSI often seek peer support online, as seen in the online NSSI support communities that have many thousands of users (e.g., over 160,000 users of the /r/selfharm subreddit on Reddit.com). Of all people with NSSI, about one‐third will seek help online (Frost and Casey 2016). Moreover, online help‐seekers tend to have more severe and more recent NSSI than those who do not seek help online (De Riggi et al. 2018; Frost and Casey 2016). Of online help‐seekers, two‐thirds report that they are actively trying to stop engaging in NSSI (Corcoran and Andover 2020).

Prosocial goals figure prominently in online help‐seeking by people with NSSI. When people seek help online for NSSI, they typically seek social support in the form of emotional support and coping strategies (Daine et al. 2013). Online NSSI help‐seekers tend to discuss more than just the specifics of NSSI; rather, they typically discuss the socioemotional contexts in which NSSI occurs (Preston et al. 2023). The animating moral concern of online NSSI communities is care, both seeking and providing (Preston et al. 2023). Young‐adult online help‐seekers retrospectively report seeking out a sense of belonging and identity (Stänicke 2023).

Alongside prosocial opportunities, online NSSI support communities carry significant risks. While social media use in general has not been shown to be associated with NSSI (Nesi et al. 2021), there is some evidence that the onset of NSSI is particularly vulnerable to social contagion, that is, the spread of a behavior within a group, which can occur online (Jarvi et al. 2013). Young‐adult users of online NSSI support communities describe an environment that facilitates risk‐taking and lacks structure and accountability, that is, there is “no one in charge” (Stänicke 2023, 160). A systematic review on the impact of viewing and sharing self‐harm‐related imagery and videos found a wide range of potential effects, including concerns about imitation, reinforcement, and normalization of NSSI (Marchant et al. 2021).

It is often unclear who bears responsibility for and what can be done about the risky aspects of online NSSI communities. In qualitative studies, users of these communities describe the burden associated with being exposed to the intense suffering of others and the desire for someone knowledgeable to intervene when suicide risk appears (Lavis and Winter 2020; Stänicke 2023). Qualitative studies further reveal that while risk and protective factors for safe use of these communities tend to focus on individual factors, platform policies and procedures can also support safety (Thorn et al. 2023). There is debate over which measures platforms should take to protect users experiencing NSSI. For example, in February 2019, Facebook and Instagram banned graphic images of self‐harm following the suicides of multiple young users of the platforms (Smith and Cipolli 2022). Following the ban, users expressed grief over the loss of these platforms as communities for social support and celebration of recovery (Smith and Cipolli 2022).

Many researchers of online NSSI communities agree that the social contagion view misses crucial aspects of the picture, and they argue for policies and platform design that promote social support and harm reduction (Alhassan et al. 2021; Lavis and Winter 2020; Preston and West 2022; Smith and Cipolli 2022; Thorn et al. 2023). Thorn et al. (2023) oppose blanket bans on NSSI content; rather, they encourage case‐by‐case assessment by moderators to avoid removal of effective social support. Preston and West (2022) promote online NSSI communities as the best setting to study the benefits of NSSI harm reduction practices since harm reduction practices like wound care are already promoted by users of online NSSI communities. Finally, scholars agree that further research is needed to guide policy and design (Lavis and Winter 2020; Preston and West 2022; Smith and Cipolli 2022; Thorn et al. 2023).

The platform that is the focus of this study differs from other online NSSI support communities in ways that make it especially valuable to study. In the studies reviewed above, the online NSSI support communities tend to be subgroups that operate on general‐use platforms like Reddit, Twitter, or Instagram. They congregate via user‐created and user‐moderated message boards on Reddit or hashtags on Twitter or Instagram. In contrast, this platform bills itself as a “mental health support community.” As a platform purpose‐built for mental health support, its design can be examined for how well it serves only that purpose, in contrast to other general‐use platforms. The platform further differs from previously studied NSSI support communities because, at the time of data collection, it required users to label their posts from a platform‐provided set of options, including a “Self Harm” label. This creates a means by which to filter for “Self Harm”‐related content. The platform also shares user birthdates with researchers, allowing for the filtering of posts to include only those by adolescents. Finally, to our knowledge, this platform is the only large‐scale platform hosting an NSSI support community that does not allow users to upload images. Disallowing images yields user content that is entirely text and ideal for study with validated natural language processing approaches. These differences make this platform valuable to study to better understand how to promote safety in online NSSI communities.

In this study, we used topic modeling to explore the content of adolescents' help‐seeking posts in the “Self Harm” category on this peer support platform. Topic modeling is an unsupervised machine learning method that takes a large body of text and produces a set of topics that characterizes the complete text. Results are examined through three lenses: (1) in context of previous findings about goals of online help‐seekers with NSSI, specifically regarding the balance of prosocial goals and risks of social contagion; (2) in comparison to other topic model‐based studies of related online communities, specifically regarding overlapping topics or themes; and (3) in consideration of the platform's purpose and design, specifically regarding how platform design choices may affect the risk of transitioning from NSSI to suicide. This study contributes to the important tasks of understanding online help‐seeking behavior and guiding policy and platform design.

## 1 Methods

### 1.1 Data Selection

Data were licensed according to the terms of the platform's Data Sharing Agreement. The Institutional Review Board at the University of Central Florida reviewed this project and made a determination of Not Human Research (IRB ID: SBE‐18‐14660; Research ID: 1066191). Data were obtained via SQL query from the platform's database in June 2021. Data were filtered to include posts that users labeled with the “Self Harm” category from users that were aged 13 to 24 at the time of posting, which was calculated from post timestamp and user‐reported date of birth. Data were excluded that had been subsequently deleted by the user or that were produced by users who subsequently deleted their account. These criteria yielded a dataset of 575,261 posts.

### 1.2 Participants

There were 114,937 unique users identified in the set of all posts. Aligning with previous research on who seeks help online for self‐injury (Frost and Casey 2016), the majority of users self‐reported “female” gender (*N* = 69,668; 60.61%). The remaining 45,269 users are approximately evenly split between “male” (*N* = 18,549; 16.14%) and “other” (*N* = 26,720; 23.25%) gender identifiers. Poster age range was calculated at the time of the first post in the “Self Harm” category. Poster age range hewed to the inclusion criteria (13–24), with a mean age of 17.5 years old (SD = 2.58 years).

### 1.3 Data Preprocessing

Following the data pre‐processing steps for topic modeling reported by Franz et al. (2020), we removed excess whitespace, punctuation, numbers, and stop words. Stop words are common terms with little unique semantic meaning, for example, “and” or “the.” To further pare down meaningless terms in the data, we filtered out words that appeared only once (e.g., misspellings, nonsense terms, dozens of permutations of “Ahhhhhhh”). Upon completion of these steps, the data retained 113,506 total discrete terms. We determined the top 20 terms (see Table 1).

**TABLE 1:** Top 20 terms by number of times used.

Term	Frequency
Feel	96,323
Cut	86,894
Life	55,088
People	46,986
Die	42,549
Anymore	41,854
Time	38,439
Hate	36,602
Talk	32,248
Cutting	32,219
Wanna	30,658
Stop	30,631
Kill	29,159
Day	29,007
Bad	28,688
Clean	26,384
Love	25,092
Pain	24,292
Fucking	23,991
Feeling	23,682

### 1.4 Post Aggregation by Author

The complete set of posts included 575,261 posts from 114,937 users. Individual posts had a mean length of 26.46 terms (SD = 51.31; median = 15), which is well below the 50‐term threshold recommended for stable, meaningful topic models (Vayansky and Kumar 2020). A similar problem arises in topic modeling studies of Twitter data, and an aggregation approach has been developed to address it (Hong and Davison 2010; Steinskog et al. 2017). The dataset for this study includes the unique user identification number, that is, author label for all posts. After aggregating posts by author, the author‐aggregated documents (*N* = 112,626) had a mean length of 132.48 terms and a median length of 37 terms. Number of posts per poster ranged from one to 3086 (mean = 5.02, SD = 21.48; median = 1).

### 1.5 Topic Model Selection

Three Latent Dirichlet Allocation (LDA; Blei et al. 2003) topic models were run with varying levels of *k*, that is, researcher specified numbers of topics. Based on previous literature on similar datasets showing interpretable topic models around 10 topics, topic models were run with eight, 10, and 12 topics, respectively. The first author assessed each topic model based on two criteria: coherence score and human interpretability. Human interpretability was assessed based on the top 20 terms of each topic. For the three models (of eight, 10, and 12 topics each), clinical expertise of the first and senior authors was used to label each topic with a theme. Models were judged based on how many of the topics were interpretable, how many topics were not interpretable, and whether the added topics captured meaning that was not already covered by other topics.

Topic modeling yielded three models of eight, 10, and 12 topics each. Coherence scores were calculated (see Table 2). Scores closer to zero denote better performance. Topic coherence scores were similar across the three models, both in terms of range and mean.

**TABLE 2:** Coherence scores for three topic models.

Topic	Model 1 (*k* = 8)	Model 2 (*k* = 10)	Model 3 (*k* = 12)
1	−104.37	−112.21	−96.80
2	−100.02	−103.50	−103.50
3	−97.60	−98.22	−101.95
4	−105.17	−91.71	−92.12
5	−117.77	−118.26	−112.98
6	−85.84	−99.13	−103.79
7	−93.32	−101.30	−97.96
8	−85.51	−90.84	−88.58
9		−103.11	−97.95
10		−94.43	−104.30
11			−123.10
12			−106.37

The top‐20 terms for each topic were identified for the three models, and themes were inferred based on the clinical expertise of the first and senior authors. Model three was selected because its topics were the most distinct from each other, and the additional topics introduced new themes (see Table 3 in Section 2; for models one and two, see Tables S1 and S2). Many themes recurred across the three models, and the 12‐topic model included the strongest themes from the 8‐ and 10‐topic models (e.g., self‐harm abstention and stay strong) while adding strong new themes (e.g., time spent crying and hiding self‐harm).

**TABLE 3:** Top‐20 terms per topic of model 3 (*k* = 12).

Topic	Terms	Theme
1	Clean, cut, days, harm, months, weeks, relapsed, urge, relapse, bad, week, day, month, tonight, stop, hard, harming, urges, blades, cutting	Self‐harm abstention
2	Love, people, kik, talk, guys, stay, strong, day, hope, beautiful, stop, message, selfharm, hey, post, person, amazing, friends, app, abused	Stay strong
3	Die, hate, kill, fucking, wanna, shit, fuck, gonna, fat, ugly, stupid, sick, ugh, talk, bad, deserve, god, hates, depressed, tonight	Explicit self‐loathing
4	Feel, people, talk, happy, time, tired, friends, depression, sad, feeling, wrong, understand, hard, lonely, friend, honestly, makes, lot, person, feels	Difficulty expressing feelings
5	Feeling, feel, depressed, suicidal, family, hurt, afraid, worse, mental, reason, sad, anxiety, bad, thinking, makes, depression, stop, parents, recently, hurting	Mental ill health
6	Life, anymore, suicide, live, care, tired, living, cares, dead, alive, world, worthless, goodbye, die, killing, ready, suicidal, bye, worth, commit	Hopeless suicide
7	Scars, cuts, arm, school, started, deep, scared, time, cut, arms, wrist, hide, cutting, told, blood, blade, found, coward, wear, bad	Hiding self‐harm
8	Cut, feel, cutting, stop, bad, wanna, idk, pain, fuck, time, scared, hurt, hurts, rn, numb, friend, broke, boyfriend, kinda, thinking	Self‐harm struggle
9	Night, cry, sleep, crying, day, time, head, smile, girl, left, eyes, wake, tears, love, bed, stay, blood, blade, fault, gonna	Time spent crying
10	Mom, told, school, dad, home, friend, parents, friends, called, sister, started, time, house, mother, guy, brother, family, people, girl, day	Family and friends
11	Skin, people, write, hurt, feel, harm, watch, time, red, play, water, start, music, safe, cut, friend, remember, burn, read, listen	Distraction from self‐harm
12	Pain, feel, mind, inside, life, heart, world, lost, broken, death, body, god, real, time, love, head, control, hope, fight, change	Philosophical thoughts

### 1.6 R Packages

Data were analyzed using *R* version 4.3.0 and *R* packages *rio* (version 0.5.29), *here* (version 1.0.1), *tidyverse* (version 2.0.0), *tidytext* (version 0.4.1), *tm* (version 0.7–11), *topicmodels* (version 0.2–14), *topicdoc* (version 0.1.1), and *beepr* (version 1.3). Execution of these methods in *R* was guided by the book, Text Mining with R by Julia Silge and David Robinson (https://www.tidytextmining.com).

## 2 Results

There were 575,261 posts by 114,937 unique users (mean age = 17.5 years, 60.61% female). A 12‐topic model was selected based on coherence scores and human interpretability. The first and senior authors analyzed the 20 top terms per topic to infer themes based on their clinical expertise (see Table 3). For example, topic one contains terms associated with resisting addictive or compulsive behavior (e.g., “clean,” “relapse,” “urge”), plus terms associated with passing time (e.g., “days,” “months,” “weeks”). Taken together, these terms likely relate to time spent resisting urges to engage in NSSI, hence the theme, “self‐harm abstention.”

Illustrative quotes from the posts are not provided for three reasons. First, following best practices for bodies of text with documents that are too brief for standard topic modeling (Hong and Davison 2010; Steinskog et al. 2017), the posts were aggregated by author (see Section 1 for details). As a result, it would be misleading to select individual posts to illustrate the topics that characterize a set of longer, more varied, author‐aggregated documents. Second, the platform that is the focus of this study is not a public, searchable, scrapable platform like Twitter or Reddit. Therefore, users of the platform may expect more privacy for the content they post. Third, and relatedly, while users of the platform do consent to share their data with researchers via the Terms of Service, users may not read the Terms of Service before consenting, rendering that consent uninformed. As such, we err on the side of caution by not publishing verbatim or paraphrased content.

The top topic was calculated for all documents, which was summarized to show which topics predominate in the corpus. Given that the most common topic focuses on suicidal content, all topics were also assessed for NSSI content and morbid/suicidal content (see Table 4).

**TABLE 4:** Top topics of author‐aggregated posts (*N* = 112,626), plus whether NSSI or morbid/suicidal content appeared in the top‐20 terms.

Rank	Topic	Frequency	Theme	NSSI content	Morbid/suicidal content
1	6	14,185	Hopeless suicide		Yes
2	4	12,164	Difficulty expressing feelings		
3	3	11,907	Explicit self‐loathing		Yes
4	1	11,487	Self‐harm abstention	Yes	
5	8	10,175	Self‐harm struggle	Yes	
6	12	9374	Philosophical thoughts		Yes
7	5	8956	Mental ill health		Yes
8	7	8397	Hiding self‐harm	Yes	
9	10	8235	Family and friends		
10	9	7562	Time spent crying	Yes	
11	2	6834	Stay strong	Yes	
12	11	3350	Distraction from self‐harm	Yes	

Of the total set of 112,626 documents, 47,805 had an NSSI‐related top topic (42.45%) while 44,422 had a morbid or suicidal top topic (39.44%).

## 3 Discussion

In this study, we set out to use topic modeling to explore the content of help‐seeking posts under the “Self Harm” category on a purpose‐built mental health peer support platform. Our goal was to contribute to the important tasks of describing online help‐seeking behavior and guiding policy and platform design. Our approach produced a 12‐topic model with interpretable themes. The themes ranged from discussion of NSSI urges, abstention, and relapse (Topic 1, “self‐harm abstention”) to angry or hopeless suicidality (Topics 3 and 6, “explicit self‐loathing” and “hopeless suicidality”) to encouragement and offers of connection (Topic 2, “stay strong”). We found that the top topics of the documents were approximately evenly split between NSSI‐related topics and morbid or suicidal topics. The topic model separated NSSI‐related terms and suicide‐related terms into different topics.

Our findings agree with most previous findings on the goals of online help‐seekers with NSSI. The presence of topics related to abstaining from NSSI, feeling conflicted about engaging in NSSI, hiding NSSI, and distracting oneself from urges to self‐injure supports the finding that most help‐seekers are actively trying to stop engaging in NSSI (Corcoran and Andover 2020). The prevalence of terms related to suffering, struggle, and hopelessness supports the finding that online help‐seekers want emotional support (Daine et al. 2013). The prevalence of these terms may also support models of NSSI that include NSSI's ability to relieve negative affect (Hooley and Franklin 2018). Finally, the severe suffering and suicidality captured by the topic model align with concerns about the burden of being exposed to the intense suffering of others and the desire for someone knowledgeable to intervene when suicide risk appears (Lavis and Winter 2020; Stänicke 2023). While the narrow range of topics, limited mostly to NSSI and suicide, may not align with findings that online NSSI help‐seekers favor discussions of the varied events, relationships, and contexts related to NSSI (Preston et al. 2023), this discrepancy may be explained by the siloing effects of requiring users to select one category for their post.

Our findings share numerous themes with topic modeling studies of online behavior and communities relevant to online help‐seeking for NSSI. While our study lacks the longitudinal component used by Feldhege et al. (2023), we found similar themes of suicidality, hopelessness, offering connection, and social support. While we focused on unigrams, that is, single terms, in contrast to the focus on trigrams by Alhassan et al. (2021), we found similar themes of self‐harm struggle or infliction and self‐harm abstention or recovery. While we selected a model with 12 topics versus the 26‐topic model selected by Preston et al. (2023), we found that there were eight similar topics that appeared in both models (hopeless suicide, explicit self‐loathing, self‐harm struggle, philosophical thoughts, mental ill health, hiding self‐harm, family and friends, and distraction from self‐harm). While Kim and Yu (2022) focused on NSSI‐related search queries, shared themes emerged, including anger and struggle, hiding self‐harm scars, and mental ill health or depression. Eleven of the 12 themes that we inferred from our topic model were mirrored in previous studies (Alhassan et al. 2021; Feldhege et al. 2023; Kim and Yu 2022; Preston et al. 2023). Only the “time spent crying” topic did not have a parallel in a previous study. A key difference between our findings and others is that we did not find topics that expressed pro‐self‐harm sentiment, nor did we find topics focused on NSSI or suicide methods, at least one of which appeared in all of the above‐mentioned studies.

Regarding the purpose and design of the platform, our findings highlight the importance of major platform characteristics. First, the platform's rules for the “Self Harm” category forbid pro‐self‐harm content and graphic descriptions of NSSI. The absence of this content from our topic model, while it appears in the topic models of other studies of online NSSI support communities, suggests that the platform's rules help to reduce this content. For example, Kim and Yu (2022) identified a topic that included queries about how to self‐injure without pain, and Preston et al. (2023) identified a topic that included specific descriptions of suicide attempts. Second, the platform required that users select a single label for each post from 33 predefined options (e.g., “Self Harm,” “Family,” “Bullying,” “LGBT,” “Depression”). The narrow focus of our topic model on NSSI and suicide, which contrasts with the varied content found by Preston et al. (2023), may indicate that the post‐labeling system creates exclusive silos that curtail the inclusion of broader socioemotional context.

The prevalence of suicide‐related topics in our topic model points to a crucial platform characteristic. Previous topic modeling studies that focused on a body of text collected around the term “self‐harm” had found topics more specifically focused on NSSI (Alhassan et al. 2021; Kim and Yu 2022; Preston and West 2023). Why then did morbid or suicidal content match the prevalence of NSSI‐related content in our results? While the platform provides a wide range of category labels for posts, they do not offer a “Suicide” label. This omission may cause those expressing suicidal thoughts and feelings to use the “Self Harm” label to identify their posts.

The conflation of NSSI and suicide may pose risks to all parties. For a suicidal young person naïve to NSSI, exposure to discussions of NSSI urges and experiences might cause the onset of NSSI. Initiation of NSSI is thought to be more vulnerable to social contagion than the recurrence of NSSI (Jarvi et al. 2013), and the use of NSSI as an anti‐suicide measure is commonly reported (Czyz et al. 2021). For a young person with NSSI who is not experiencing suicidal ideation, concerns loom about suicide clustering and the burden of supporting people with intense suffering while trying to manage one's own challenges (Lavis and Winter 2020; Swedo et al. 2021).

Numerous aspects of this study strengthen its findings. This study examines the help‐seeking behavior of over 100,000 adolescents, surpassing the scale of qualitative studies and improving on the specificity of quantitative studies that lack age‐related information, for example, Reddit‐based studies (Kim et al. 2023; Stänicke 2023). The naturalistic, observational quality of this study's data provides important convergent evidence to integrate with previous interview‐ or questionnaire‐based studies of online help‐seeking and social support. Topic modeling is a powerful data‐driven approach that is underused in clinical science. This approach is more common in computer science, and it is a particular strength of this study that the development and interpretation of the topic model benefited from clinical expertise.

The study also had important limitations. First, as many scholars have pointed out (e.g., Hagg et al. 2022), topic modeling is rife with subjectivity and researcher determined degrees of freedom. For example, the pre‐processing of a corpus of text for topic modeling requires multiple decisions about the inclusion and exclusion of terms and documents (e.g., choice of stopword list) that can be idiosyncratic. Second, the interpretation of topic models calls on close reading skills from literature class alongside more evidence‐based clinical skills. Third, we have not explored whether morbid/suicidal content emerges in other post categories like it does in the “Self Harm” category. Finally, the dataset was collected in 2021, and the platform has changed important aspects of the user experience since then (e.g., users are no longer required to select a category when they post).

Despite these limitations, this study has important implications. This study reveals the immense suffering expressed by adolescents seeking help online for self‐harm. The themes revealed in our topic model mostly align with previous findings about the goals of online NSSI help‐seekers and the discourse in online NSSI communities. The differences that emerged may point to important design considerations for online mental health support communities: rules probably help, categories may create silos, and not offering a “Suicide” label may increase risk.

Future directions grow out of limitations and implications. Synthesizing topic model findings across studies would be more meaningful if research teams used similar pre‐processing methods and transparent reporting (Hagg et al. 2022). To test our speculation about the effect of omitting a “Suicide” category, further investigation should explore whether platforms that limit suicidal expressions see suicidality bubble up in unintended places. Implications regarding platform design support three takeaways. Platforms should implement safety‐focused rules, attend to unintended outcomes of requiring user categorization of content, and grapple with their responsibility to people seeking help for both self‐injurious and suicidal thoughts and behaviors.

## 4 Conclusion

We used topic modeling to explore the content of adolescent help‐seeking expressions under the “Self Harm” category on a purpose‐built online peer support platform. Our results mostly aligned with previous studies. Important exceptions included relatively less specific situational narrative, absence of pro‐self‐harm sentiment and specific descriptions of self‐harm methods, and the high prevalence of morbid/suicidal content. Each of these exceptions points to a design feature of the platform that may explain the findings. The platform's category labels may silo content more than other platforms; the platform's rules may reduce content that can contribute to social contagion; and the platform's omission of a “Suicide” category may funnel suicidal users into the “Self Harm” category. We hope that future research will investigate the effects of these features, and we encourage online mental health platforms to work with researchers in pursuit of this goal.

## Author Contributions

**Monika Neff Lind:** conceptualization (lead), formal analysis (lead), methodology (lead), writing – original draft (lead), writing – review and editing (lead). **Afsaneh Razi:** conceptualization (equal), formal analysis (equal), methodology (equal), writing – review and editing (equal). **Hanneke Scholten:** conceptualization (equal), methodology (equal), writing – review and editing (equal). **Madeleine J. George:** conceptualization (equal), methodology (equal), writing – review and editing (equal). **Munmun De Choudhury:** conceptualization (equal), methodology (equal), writing – review and editing (equal). **Isabela Granic:** conceptualization (equal), methodology (equal), writing – review and editing (equal). **Shalini Lal:** conceptualization (equal), methodology (equal), writing – review and editing (equal). **Pamela J. Wisniewski:** conceptualization (equal), methodology (equal), writing – review and editing (equal). **Nicholas B. Allen:** conceptualization (equal), methodology (equal), writing – review and editing (equal).

## Ethics Statement

The Institutional Review Board at the University of Central Florida reviewed this project and made a determination of Not Human Research (IRB ID: SBE‐18‐14660; Research ID: 1066191).

## Conflicts of Interest

Monika Neff Lind and Nicholas B. Allen have an equity interest in Ksana Health Inc. No Ksana Health services or products were used in the current project.

## Supporting information


**Tables S1–S2:** sltb70055‐sup‐0001‐TableS1‐S2.docx.

## Data Availability

The data that support the findings of this study are available from the online peer support platform. Restrictions apply to the availability of these data, which were used under license for this study.
