# Supplementary material for: When “Self‐Harm” Means “Suicide”: A Topic Modeling Study of Adolescent Online Help‐Seeking for Self‐Harm
Source: Suicide Life Threat Behav. 2025 Nov 17;55(6):e70055. doi: 10.1111/sltb.70055 (PMC12621541; doi:10.1111/sltb.70055)
Supplement: Supplementary file 1 — Tables S1–S2: sltb70055‐sup‐0001‐TableS1‐S2.docx. [file SLTB-55-0-s001.docx]

**Supplementary Information**

Supplementary Table 1. Top-20 terms per topic of model 1 (*k* = 8); topics from which themes do not emerge are labeled by that topic’s top-three terms

| Topic | Terms | Theme |
| --- | --- | --- |
| 1 | hurt, skin, pain, blood, harm, feel, people, time, body, red, write, start, trigger, watch, hand, cut, mind, thinking, feeling, scars | Self-harm body |
| 2 | people, love, guys, day, stay, hope, beautiful, strong, friends, person, stop, feel, god, talk, post, life, happy, hey, selfharm, app | Stay strong |
| 3 | hate, wanna, die, talk, fucking, kill, shit, fuck, kik, gonna, sleep, depressed, fat, bad, idk, feel, hurt, ugly, cry, stupid | Explicit self-loathing |
| 4 | time, mom, told, school, day, heart, left, girl, dad, home, friend, night, crying, eyes, smile, friends, head, inside, broken, cry | *Time, mom, told* |
| 5 | feel, feeling, depression, people, family, afraid, sad, mind, anxiety, mental, pain, understand, makes, worse, hard, depressed, life, suicidal, person, day | Mental ill health |
| 6 | life, anymore, suicide, tired, die, care, live, people, feel, happy, world, cares, living, dead, alive, kill, friends, goodbye, worthless, suicidal | Hopeless suicide |
| 7 | clean, harm, scars, months, days, cuts, weeks, cut, school, week, time, started, relapsed, bad, relapse, ago, day, night, urge, told | Self-harm abstention |
| 8 | cut, feel, cutting, stop, bad, time, pain, days, hard, blade, feeling, tonight, fuck, hurts, scared, numb, hurt, deep, razor, boyfriend | Self-harm struggle |

Supplementary Table 2. Top-20 terms per topic of model 2 (*k* = 10); topics from which themes do not emerge are labeled by that topic’s top-three terms

| Topic | Terms | Theme |
| --- | --- | --- |
| 1 | hurt, harm, people, skin, feel, time, write, body, start, attention, trigger, watch, pain, red, play, burn, remember, thinking, music, water | Distraction from self-harm |
| 2 | love, talk, people, kik, guys, stay, strong, day, hope, beautiful, stop, person, message, post, friends, hey, selfharm, app, amazing, happy | Stay strong |
| 3 | hate, die, wanna, fucking, kill, fuck, gonna, shit, talk, fat, ugly, idk, cry, stupid, crying, sleep, bad, rn, scared, hurt | Explicit self-loathing |
| 4 | feel, feeling, people, tired, time, sad, happy, hard, talk, hurt, makes, wrong, sleep, friends, understand, feels, lonely, bad, depressed, sick | Expressing feelings |
| 5 | depression, suicidal, anxiety, afraid, mental, family, depressed, feeling, worse, hospital, feel, bad, pain, taking, times, thinking, eating, day, stop, suicide | Mental ill health |
| 6 | life, anymore, suicide, live, die, care, living, world, cares, dead, alive, worthless, goodbye, people, kill, killing, ready, tired, leave, death | Hopeless suicide |
| 7 | clean, harm, scars, months, cuts, days, weeks, relapsed, relapse, week, urge, time, harming, ago, night, arm, month, day, bad, urges | Self-harm abstention |
| 8 | cut, cutting, stop, bad, days, feel, blade, tonight, time, deep, clean, pain, wrist, razor, friend, scared, boyfriend, cuts, blades, badly | Self-harm struggle |
| 9 | pain, heart, head, inside, love, smile, day, eyes, broken, cry, tears, time, mind, night, world, blood, girl, left, body, fall | *Pain, heart, head* |
| 10 | school, mom, told, friends, friend, dad, home, parents, time, started, people, called, girl, day, sister, family, house, brother, guy, mother | Family and friends |
